# Embolic Material Migration as the Predominant Contributing Factor to Prognostic Deterioration Following Combined Tumor Resection and Preoperative Embolization

**DOI:** 10.7759/cureus.57315

**Published:** 2024-03-31

**Authors:** Ryosuke Suzuki, Taisuke Akimoto, Shigeta Miyake, Yu Iida, Wataru Shimohigoshi, Yasunobu Nakai, Nobuyuki Shimizu, Katsumi Sakata, Tetsuya Yamamoto

**Affiliations:** 1 Department of Neurosurgery, Yokohama City University Graduate School of Medicine, Yokohama, JPN; 2 Department of Neurosurgery, Yokohama Brain and Spine Center, Yokohama, JPN; 3 Department of Neurosurgery, Yokohama City University Medical Center, Yokohama, JPN

**Keywords:** intracranial tumor, embolic material migration, complication, tumor resection, preoperative embolization

## Abstract

Introduction

Preoperative embolization can potentially facilitate surgical resection of challenging tumors in the intracranial and facial regions; however, its clinical efficacy remains controversial, mainly due to potential morbidity risks. We explored negative factors of the combined treatment of preoperative embolization and tumor resection that affect neurological prognosis.

Method

This retrospective study used clinical data from 132 consecutive tumors that underwent combined treatment at multiple facilities between January 2016 and May 2021. Basic patient information, tumor characteristics, and treatment details were assessed to identify predictors of deterioration as measured using the modified Rankin scale (mRS) score at three months post-treatment.

Results

Among the 126 eligible combined treatments, a deterioration in the postoperative mRS score was observed in 19/126 (15.1%). Complications related to embolization and tumor resection occurred in 8/126 (6.3%) and 19/125 (15.2%) of procedures, respectively. Multivariate analyses indicated significant associations between migration of embolic material (adjusted odds ratio 13.80; 95% confidence interval 1.25-152.52; p=0.03), elevated intraoperative blood loss (p=0.04), and deterioration of postoperative mRS score. Embolic material migration was identified as the primary prognostic factor for the deterioration of score. An analysis of 192 procedures, excluding those that exclusively used coils, identified embolization targeting the accessory meningeal artery (p=0.046) and the third segment of the internal maxillary artery (p=0.03) as a risk factor for embolic material migration.

Conclusions

Embolic material migration is the predominant factor associated with declining neurological outcome that persists into the chronic phase after combined treatment. Given that preoperative embolization is a supplementary treatment option, a thorough understanding of vascular anatomy and striving safe procedure are critical.

## Introduction

Preoperative embolization is an adjunctive treatment option for large, firm, highly vascularized intracranial and facial tumors that are challenging to remove [1-4]. Preoperative embolization theoretically leads to less intraoperative bleeding, improved surgical visibility, tumor softening, and reduced operating duration [5,6]. Tumors requiring preoperative embolization present inherent difficulties for surgical intervention, and combined treatment could reduce morbidity from resection [7]. Additionally, a recent investigation has revealed the effectiveness of preoperative embolization in postponing the recurrence of meningiomas [8]. However, several studies have raised concerns about its therapeutic value, mainly due to the morbidity associated with the procedure [9-11]. Unexpected complications from embolization may directly worsen outcomes and affect patient prognosis, regardless of surgical resection. Presently, the clinical efficacy of preoperative embolization remains controversial.

Advancements in endovascular devices and techniques have made previously inaccessible lesions treatable, potentially broadening the indication of preoperative embolization. Yet, as preoperative embolization is mainly regarded as a supplemental therapy, it is imperative to carefully weigh its benefits against the risks. Several factors linked to embolization-associated complications, important for minimizing risks, have been reported [12-14]. However, these predictors are not specific enough, limiting their utility in guiding tailored treatments in clinical practice. Additionally, few studies have thoroughly assessed risk factors during embolization that could heighten complication risks. In this study, we examined cases of neurological decline after combined treatment, seeking to pinpoint specific factors predicting worsened prognosis.

## Materials and methods

Study design and patient selection

This retrospective cohort study was conducted using clinical data from a multicenter observational registry to investigate outcomes of endovascular therapy. We included all intracranial and facial tumors planned for preoperative embolization and tumor resection from January 2016 to May 2021 at three participating facilities. Embolization strategies were set through discussions between attending surgeons and interventional neuroradiology instructors at each facility. Facilities independently decided the interval from embolization to resection, as well as surgical approaches. No exclusion criteria were established for patient age, tumor histology, target vessel, embolization technique, or resection procedure. Procedures for recurrent tumors were also included. We excluded procedures with less than three months of follow-up. Institutional review board approval of each participating facility was obtained (approval no. B200500004, B210500007, and 142100503). The need for written informed consent from patients was waived due to the retrospective nature of this study, and an opportunity to opt out was provided.

Data collection

We collected basic patient and tumor characteristics including age, sex, body mass index, comorbidities, modified Rankin scale (mRS) score on admission, maximum tumor diameter, tumor location, and pathology. Tumor locations were categorized as intradural extra-axial, intradural intra-axial, or extradural. Meningiomas were not classified according to the World Health Organization grade. Cancers and lymphomas were treated collectively as malignant tumors. We evaluated operation duration, residual tumor stain after embolization, estimated blood loss during resection, degree of resection, complications related to embolization (including symptomatic thromboembolism, hemorrhagic event, migration of embolic materials, and symptomatic puncture site event), and resection (including hemorrhagic event, symptomatic ischemic infarction, and cranial nerve deficit) from medical records and radiographical data. To assess the treatment impact of combined treatment on patient prognosis, we compared preoperative and three-month postoperative mRS scores. Embolization specifics, such as anesthesia type, pharmacological provocation test use, target feeders, embolic materials (including Embosphere®, Merit Medical Systems, South Jordan, UT, USA, n-butyl-2-cyanoacrylate, and detachable or pushable coil), distance from the microcatheter tip to the tumor when embolic material was injected (<10 mm, 10-30 mm, or >30 mm), and post-embolization angiographic results, were collected.

Statistical analysis

Quantitative variables are presented as mean ± standard error, and categorical valuables are presented as frequency or percentages. The participants were stratified into the deteriorated or non-deteriorated group two groups based on mRS scores changes. Between-group differences were analyzed using the chi-squared test or Fisher’s exact test for categorical variables and using the Wilcoxon test for continuous variables. A multivariate logistic regression analysis identified predictive factors for postoperative mRS score deterioration, including only variables with a p-value of <0.05 from univariate analysis. A p-value of <0.05 was considered statistically significant. All statistical analyses were conducted using JMP Pro 15 (SAS Institute Inc., Cary, NC, USA).

## Results

Clinical characteristics and treatment outcomes of all procedures

Between January 1, 2016, and May 31, 2021, our group conducted 132 combined treatments for intracranial and facial tumors at three facilities. Six procedures were excluded due to insufficient data (five procedures) and an unrelated fatality before evaluating the outcome (one procedure), resulting in 126 treatments enrolled in this study. Table 1 details the clinical characteristics and treatment outcomes of all procedures. The mean age of patients was 57.5 ± 1.4 years, and 56/126 (44.4%) were male. Overall, 93/126 (73.8%) cases were symptomatic, while 119/126 (94.4%) exhibited mRS score of ≤2 on admission. The average maximum tumor diameter was 41.8 ± 1.4 mm, with no statistically significant difference between the groups (41.9 ± 1.5 mm vs. 41.3 ± 3.6 mm; p=0.41). Tumor locations were 97/126 (77.0%) intradural extra-axial tumors, 15/126 (11.9%) intra-axial tumors, and 14/126 (11.1%) extradural or facial tumors. Meningiomas constituted 87/126 (69.0%) cases, hemangioblastomas 15/126 (11.9%) cases, solitary fibrous tumors 8/126 (6.3%), and malignant tumors (cancer or lymphoma) 7/126 (5.6%) cases. The average embolization and resection duration were 110.7 ± 3.4 minutes and 8.0 ± 0.4 hours, respectively. A reduction of more than 60% in angiographic tumor stain was achieved in 70/126 (55.6%) of the procedures. The mean estimated blood loss was 362.6 ± 43.9 mL, with complete resection in 88/126 (69.8%) cases. Three months after combined treatment, 118/126 (93.7%) maintained an mRS score below 2, while 19/126 (15.1%) worsened their mRS scores.

**Table 1:** Demographics and clinical characteristics of all cases mm, millimeter; mRS, modified Rankin scale score; SE, standard error

	Total (n=126)
Age, mean ± SE (years)	57.5 ± 1.4
Male, n (%)	56 (44.4)
Comorbidity, n (%)	
Hypertension	44 (34.9)
Dyslipidemia	22 (17.5)
Diabetes mellitus	11 (8.7)
Chronic kidney disease	25 (19.8)
Antiplatelet or anticoagulant use, n (%)	11 (8.7)
Symptom, n (%)	93 (73.8)
Neurological deficit	60 (47.6)
Elevated intracranial pressure	14 (11.1)
Epilepsy	10 (7.9)
Hemorrhagic event	9 (7.1)
Maximum tumor diameter, mean ± SE (mm)	41.8 ± 1.4
Tumor location, n (%)	
Intradural extra-axial tumor	97 (77.0)
Intradural intra-axial tumor	15 (11.9)
Extradural or facial tumor	14 (11.1)
Tumors located at the skull base, n (%)	44 (34.9)
Pathology, n (%)	
Meningioma	87 (69.0)
Hemangioblastoma	15 (11.9)
Solitary fibrous tumor	8 (6.3)
Malignant tumor (cancer or lymphoma)	7 (5.6)
Glomus tumor	1 (0.7)
Embolization duration, mean ± SE (minutes)	110.7 ± 3.4
Angiographic stain reduction after embolization, n (%)	
<30%	26 (20.6)
30% to 59%	30 (23.8)
≥60%	70 (55.6)
Interval from embolization to resection, mean ± SE (days)	2.2 ± 0.3
Tumor resection duration, mean ± SE (hours)	8.0 ± 0.4
Intraoperative blood loss, mean ± SE (ml)	362.6 ± 43.9
Complete resection, n (%)	88 (69.8)
mRS ≤ 2 on admission, n (%)	119 (94.4)
mRS ≤ 2 three months after combined treatment, n (%)	118 (93.7)
Deterioration of mRS after combined treatment, n (%)	19 (15.1)

Perioperative complications related to the combined treatment

Table 2 outlines the perioperative complications from 126 procedures. Embolization-related complications were observed in 8/126 (6.3%) cases, comprising one case of symptomatic thromboembolism, one case of asymptomatic bleeding due to vessel perforation, and six cases of embolic material migration (leading to three permanent and two transient cases of cranial nerve palsy, and one case of permanent hemiplegia). Embolization-related complications occurred more frequently in the deteriorated group compared to the non-deteriorated group (5/19 [26.3%] vs. 3/107 [2.8%]). In one patient with embolic material migration, surgery was canceled due to severe visual field impairment. Nineteen complications associated with resection occurred in 18 cases (one patient in the deteriorated group experienced multiple complications), including two cases of intraoperative hemorrhage requiring additional surgery, six cases of symptomatic brain infarction, and 12 cases of cranial nerve palsy. The deteriorated group exhibited a higher frequency of resection-related complications (8/18 [44.4%]) than the non-deteriorated group (11/107 [10.3%]).

**Table 2:** Complication related to preoperative embolization and tumor resection *One patient had removal canceled due to complications from tumor embolization †Includes one deteriorated mRS case with multiple complications mRS, modified Rankin scale score

	Total	Deterioration of mRS	
		No (n=107)	Yes (n=19)
Complication related to embolization, n (%)	8 (6.3)	3 (2.8)	5 (26.3)
Symptomatic thromboembolism	1 (0.7)	0	1 (5.3)
Asymptomatic vessel perforation	1 (0.7)	1 (0.9)	0
Migration of embolic materials	6 (4.7)	2 (1.9)	4 (21.1)
Permanent sequelae			
Visual field impairment	1 (0.7)	0	1 (5.3)
Facial numbness	2 (1.6)	0	2 (10.5)
Hemiplegia	1 (0.7)	0	1 (5.3)
Transient sequelae			
Visual disturbance	1 (0.7)	1 (0.9)	0
Diplopia	1 (0.7)	1 (0.9)	0
Symptomatic puncture site complication	0	0	0
Postoperative intratumoral hemorrhage	0	0	0
Complication related to tumor resection, n (%)*^, †^	19 (15.2)	11 (10.3)	8 (44.4)
Postoperative hemorrhage requiring re-operation	2 (1.6)	1 (0.9)	1 (5.5)
Symptomatic brain infarction	6 (4.8)	2 (1.9)	4 (22.2)
Cranial nerve palsy	12 (9.6)	8 (7.5)	4 (22.2)
Mortality	0	0	0

Prognostic factors for postoperative deterioration in mRS score

Table 3 presents the analysis of prognostic factors for postoperative mRS score deterioration. Comparing the deteriorated and non-deteriorated groups, significant differences were found in the proportion of non-intradural extra-axial tumors (p=0.01), the rate of non-meningioma pathologies (p=0.03), embolization duration (p=0.01), the frequency of embolic material migration (p=0.004), intraoperative blood loss (p=0.001), and the incidence of symptomatic brain infarction from resection (p=0.003). Multivariate analysis confirmed that embolic materials migration (p=0.03) and increased intraoperative blood loss (p=0.04) were significant risk factors for deterioration in postoperative mRS score. Additionally, embolic material migration was identified as the paramount prognostic factor (adjusted odds ratio 13.80; 95% confidence interval 1.25-152.52; p=0.03).

**Table 3:** Univariate and multivariate analysis of postoperative mRS deterioration *The p-value is considered significant †One patient had removal canceled due to complications from tumor embolization CI, confidence interval; mm, millimeter; mRS, modified Rankin scale score; N/A, not applicable; OR, odds ratio; SE, standard error

	Deterioration of mRS		Univariate analysis	p-value	Multivariate analysis	p-value
	No (n=107)	Yes (n=19)	OR (95% CI)		OR (95% CI)	
Patient characteristics						
Age (years), mean ± SE	57.7 ± 1.5	56.4 ± 3.5	0.99 (0.96-1.03)	0.72	N/A	N/A
Male, n (%)	48 (44.9)	8 (42.1)	0.89 (0.33-2.40)	0.82	N/A	N/A
Symptomatic lesion, n (%)	78 (72.9)	15 (78.9)	1.39 (0.43-4.55)	0.58	N/A	N/A
Tumor characteristics						
Maximum diameter (mm), mean ± SE	41.9 ± 1.5	41.3 ± 3.6	1.00 (0.97-1.03)	0.87	N/A	N/A
Other than intradural extra-axial tumor, n (%)	20 (18.7)	9 (47.4)	3.92 (1.41-10.89)	0.01*	6.93 (0.42-113.88)	0.18
Pathology, n (%)						
Meningioma	78 (72.9)	9 (47.4)	0.33 (0.12-0.91)	0.03*	0.90 (0.06-14.09)	0.95
Hemangioblastoma	12 (11.2)	3 (15.8)	1.48 (0.38-5.85)	0.57	N/A	N/A
Solitary fibrous tumor	7 (6.5)	1 (5.3)	0.79 (0.09-6.84)	0.83		
Malignant tumor (cancer and lymphoma)	5 (4.7)	2 (10.5)	2.40 (0.43-13.38)	0.32	N/A	N/A
Factors related to preoperative embolization						
Embolization duration (min), mean ± SE	106.4 ± 3.6	134.6 ± 8.5	1.02 (1.01-1.03)	0.01*	1.01 (1.00-1.03)	0.15
Intracranial target feeder, n (%)	14 (13.1)	2 (10.5)	0.78 (0.16-3.75)	0.76	N/A	N/A
Complication, n (%)	3 (2.8)	5 (26)	N/A	N/A	N/A	
Symptomatic thromboembolism	0	1 (5.3)	N/A	N/A	N/A	N/A
Embolic materials migration	2 (1.9)	4 (21.1)	14.00 (2.36-83.14)	0.004*	13.80 (1.25-152.52)	0.03*
Factors related to tumor resection^†^	11 (10.3)	8 (44.4)	N/A	N/A	N/A	N/A
Resection duration (hour), mean ± SE	7.8 ± 0.4	9.2 ± 0.9	1.09 (0.97-1.23)	0.17	N/A	N/A
Intraoperative blood loss (ml), mean ± SE	286.0 ± 44.0	818.1 ± 107.4	1.00 (1.00-1.00)	0.001*	1.00 (1.00-1.00)	0.04*
Complete resection, n (%)	76 (71.0)	12 (66.7)	0.82 (0.28-2.37)	0.71	N/A	N/A
Complication, n (%)	11 (10.3)	8 (44.4)	N/A	N/A	N/A	N/A
Postoperative hemorrhage requiring reoperation	1 (1.0)	1 (5.6)	6.24 (0.37-104.47)	0.20	N/A	N/A
Symptomatic brain infarction	2 (1.9)	4 (22.2)	15.00 (2.51-89.54)	0.003*	7.11 (0.46-108.76)	0.16
Cranial nerve palsy	8 (7.5)	4 (22.2)	3.54 (0.94-13.29)	0.06	N/A	N/A

Related factors for embolic material migration

After identifying the embolic material migration as a paramount prognostic factor, further analysis was conducted to explore risk factors for this adverse event. Out of 125 embolizations (215 procedures), embolic material migration occurred in six (Table 4). Excluding 23 procedures using solely coils due to their rare migration incidents, we assessed 192 procedures (Table 5). No migration experienced in procedures targeting intracranial feeding arteries from the internal carotid artery (ICA) or vertebrobasilar artery. Among external carotid branches, the middle meningeal artery was most targeted (95/192 [49.5%]), followed by the occipital artery (16/192 [8.3%]), accessory meningeal artery (AMA) (12/192 [6.3%]), and third segment of the internal maxillary artery (IMA) (10/192 [5.2%]). Particle and liquid embolic materials were used in 89/192 (46.4%) and 103/192 (53.6%) of all procedures, respectively. The proportion of embolization targeting the AMA and third segment of the IMA was significantly higher in procedures with embolic material migration (p=0.046 and p=0.03, respectively). Factors such as the type or composition of embolic materials, the distance from the microcatheter tip to the tumor when injecting embolic material, and post-embolization angiographic results were not significantly associated with embolic material migration.

**Table 4:** Details of embolization procedures in cases with embolic material migration *The procedure that caused embolic material migration in each case AMA, accessory meningeal artery; F, female; IMA, internal maxillary artery; M, male; mm, millimeter; MMA; middle meningeal artery; NBCA, n-butyl-2-cyanoacrylate

Case	Age/sex	Tumor			Embolization procedure					
		Location	Pathology	Maximum diameter (mm)	Anesthesia	Duration (minutes)	Target vessels and used embolic materials	Stain reduction	Complication-related symptom	Prognosis of symptom
1	39/M	Intradural extra-axial	Meningioma (petroclival)	40.9	Local	75	AMA (Embosphere 100-300 µm + coil)*	< 30%	Diplopia	Temporary
2	21/F	Extradural	Organizing hematoma (paranasal sinus)	44.1	Local	125	1st session: the third segment of IMA (17% NBCA); 2nd session: the third segment of IMA (17% NBCA)*; 3rd session: the third segment of IMA (coil)	30-60%	Facial hypoesthesia	Permanent
3	49/F	Extradural	Atypical cell proliferation (intraorbital)	23.9	Local	58	1st session: the third segment of IMA (coil); 2nd session: the third segment of IMA (20% NBCA)*	< 30%	Facial hypoesthesia	Permanent
4	66/M	Intradural extra-axial	Meningioma (parasagittal)	24.0	Local	125	1st session: MMA (Embosphere 300-500 µm + coil); 2nd session: MMA (Embosphere 300-500 µm + coil)*	> 60%	Visual loss	Permanent
5	44/M	Intradural extra-axial	Meningioma (falx)	30.7	Local	93	MMA (Embosphere 300-500µm + coil)*	> 60%	Visual field impairment	Temporary
6	50/M	Intradural extra-axial	Meningioma (sphenoid ridge)	66.8	Local	236	1st session: AMA (Embosphere 100-300 µm); 2nd session: AMA (20% NBCA)*; 3rd session: MMA (17% NBCA); 4th session: the third segment of IMA (17% NBCA + coil)	30-60%	Hemiparesis	Permanent

**Table 5:** Characteristics of embolization procedures in cases with embolic material migration *The p-value is considered significant CI, confidence interval; ECA, external carotid artery; ICA, internal carotid artery; ILT, inferolateral trunk; IMA, internal maxillary artery; MHT, meningohypophyseal artery; NA, not analyzed; NBCA, n-butyl-2-cyanoacrylate; OR, odds ratio; SE, standard error

	Embolic material migration		OR (95% CI)	p-value
	Yes (n=6)	No (n=186)		
Pharmacological provocation test, n (%)	2 (33.3)	36 (19.4)	2.08 (0.37-11.82)	0.34
Target feeder, n (%)				
ICA branch				
ILT or MHT	0	3 (1.6)	-	1.00
Vertebrobasilar branch				
Superior cerebellar artery	0	11 (5.9)	-	1.00
Anterior inferior cerebellar artery	0	4 (2.2)	-	1.00
Posterior inferior cerebellar artery	0	7 (3.8)	-	1.00
ECA branch				
Middle meningeal artery	2 (33.3)	93 (50.0)	0.50 (0.09-2.80)	0.68
Occipital artery	0	16 (8.6)	-	1.00
Accessory meningeal artery	2 (33.3)	10 (5.4)	8.80 (1.44-53.93)	0.046*
Third segment of IMA	2 (33.3)	8 (4.3)	11.12 (1.77-70.00)	0.03*
First and second segments of IMA	0	9 (4.8)	-	1.00
Ascending pharyngeal artery	0	6 (3.2)	-	1.00
Distance from microcatheter tip to tumor, n (%)				
<10 mm	2 (33.3)	54 (29.0)	1.22 (0.22-6.87)	1.00
10-30 mm	2 (33.3)	96 (51.6)	0.47 (0.08-2.62)	0.44
>30 mm	2 (33.3)	36 (19.4)	2.08 (0.37-11.8)	0.34
Used embolic material, n (%)				
Particle embolic material	3 (50)	86 (45.1)	1.16 (0.23-5.91)	1.00
Embosphere 100-300 μm	1 (16.7)	41 (22.0)	0.71 (0.08-6.23)	1.00
Embosphere 300-500 μm	2 (33.3)	45 (24.2)	1.57 (0.28-8.84)	0.64
Liquid embolic material	3 (50.0)	100 (54.3)	0.86 (0.17-4.37)	1.00
<17 % NBCA	1 (16.7)	60 (32.3)	0.42 (0.05-3.67)	0.67
18-33 % NBCA	2 (33.3)	39 (21.0)	1.88 (0.33-10.67)	0.61
>33 % NBCA	0	1 (0.5)	-	1.00
Angiographic result after embolization, n (%)				
Only feeder occlusion	2 (33.3)	39 (21.0)	1.88 (0.33-10.67)	0.61
No residual stain via target vessel	3 (50.0)	91 (48.9)	1.04 (0.21-5.31)	1.00

## Discussion

This research has identified the migration of embolic material during preoperative embolization and the increased intraoperative blood loss encountered in tumor resection as significant predictors of deteriorated mRS score after combined therapy. Notably, embolic material migration was the most significant factor affecting patient outcomes.

This study primarily examined the safety aspect of combined therapy for intractable hypervascularized tumors. We found postoperative mRS score deterioration in 19 (15.1%) of 126 cases, which aligns with findings from a recent matched cohort study on preoperative embolization for large intracranial meningioma that reported a 15.2% decline [7]. Conversely, 31.8% of patients in that study’s non-embolization cohort experienced worsened mRS score postoperatively, suggesting that tumors eligible for preoperative embolization are inherently difficult to treat. Our investigation also highlights that increased intraoperative blood loss was associated with deterioration in postoperative mRS score. Excessive intraoperative bleeding, which not only leads to critical blood loss but also impairs surgical field visibility, might contribute to the deterioration by complicating the preservation of essential anatomy. Inadvertent damage to cranial nerves and major vessels can result from efforts to control bleeding. Thus, preoperative embolization could offer additional, previously unrecognized benefits by potentially enhancing neurological function preservation during tumor resection beyond its known advantages such as reducing blood loss, softening tumors, and shortening operation duration.

To our knowledge, this study is the first to underscore the significant impact of embolic material migration during preoperative embolization on postoperative deterioration of neurological function in patients receiving combined treatment. As preoperative embolization is essentially supportive treatment, meticulous attention to minimize complications is imperative during procedure. Within our study cohort, significant embolization-related complications were observed in 6.3% of procedures, aligning with recent studies [9,10,14,15]. Specifically, migration of embolic material occurred in 4.7% of our procedures. The acceptability of this complication rate is uncertain, with most reports not differentiating it from ischemic complications or cranial nerve palsy [10,14,16,17]. Notably, four out of six cases with embolic material migration led to permanent sequelae, implying a high risk of irreversible neurological symptoms due to arterial obstruction by migrated material [18]. While some physical and neurological symptoms may resolve spontaneously, our results indicate that such complications could have a lasting negative impact throughout the postoperative period. Conversely, the complications related to tumor resection, including symptomatic brain infarction and cranial nerve palsy, were not significantly linked to mRS score deterioration postoperatively. This discrepancy could be due to the limited number of participants with worsened mRS score, resulting in low statistical power. Furthermore, postoperative mRS score might have remained unchanged due to preexisting neurological impairments despite the emergence of new symptoms from the resection.

Analyses of procedures revealed a link between embolization targeting the AMA and the third segment of the IMA, and the migration of embolic materials. Several clinical factors associated with complications after preoperative embolization have been reported, such as targeting vessels other than the external carotid artery, using liquid or small particle embolic agents, and exhibiting pathological features distinct from meningiomas [12-14]. Yet, few studies have focused on the migration of embolic materials or conducted statistical analyses of the associated risk factors. Rosen et al. noted an association between the types of embolized vessels and postprocedural complications, identifying the ascending pharyngeal artery, the meningohypophyseal trunk of the ICA, and the AMA as the three targets most associated with significant risks of permanent neurological sequelae (13.0%, 11.8%, and 10.5%, respectively) [18]. Our study showed that beyond the AMA, the third segment of the IMA is associated with the embolic material migration. The tributaries of both the AMA and the third segment of IMA not only supply the nerves of the middle cranial fossa and infratemporal fossa but also possess numerous potential anastomoses with the branches of the ICA, requiring vigilant embolization [19-23]. Similarly, arterial branches supplying the cranial fossa often present clinically important anastomoses, and tributaries passing through foramina may also nourish cranial nerves. Particularly, dangerous anastomoses require constant attention, as they are always present yet inherently difficult to detect. Our research indicated that differences in the choice between particle and liquid embolic agents, particle size, the distance from microcatheter tip to tumor during embolization, and the use of pharmacological provocation tests exert no significant influence on embolic material migration. Nevertheless, liquid or small particle embolic agents always have the risk of accidental migration. Our findings emphasize the importance for practitioners to carefully select target feeders and consistently recognize the possibility of embolic material migration, regardless of the embolization technique or devices used.

Several limitations must be considered due to the retrospective multi-institutional nature of the present research and the limited sample size. A limited number of cases where postoperative deterioration in the mRS score was observed diminished the statistical power for the analysis of factors associated with poor prognosis. This concern also applies to the analysis of the migration of embolic materials. Furthermore, regarding complications, it may not always be clear whether cerebral infarctions and any neurological damage stem from preoperative embolization or tumor resection. However, a neurological evaluation was routinely conducted after preoperative embolization, aiding in the identification of any symptomatic complications induced by embolization before resection. The absence of standardized criteria for indications for combined treatment, particularly given the coexistence of various tumors with different pathological features and locations, could have influenced patient prognosis in the chronic phase. Conversely, given that outcomes were evaluated three months after treatment, the impact of tumor pathology on the worsening of mRS scores is presumed to be limited. In fact, deterioration in mRS score was observed due to disease progression in only one lymphoma case. In addition, the involvement of multiple surgeons and a lack of a standardized treatment protocol might have introduced potential bias in treatment outcomes based on individual or institutional proficiency. Our study did not include a comparison of treatment outcomes with or without preoperative embolization; therefore, the validity of supporting preoperative embolization as a standard therapeutic option for difficult-to-treat tumors remains uncertain. Although technical advancements and device innovations are broadening the applicability of embolization, it is wise to adopt a cautious approach considering the risks of complications, awaiting the results of large-scale prospective studies to establish consensus on therapeutic indications.

## Conclusions

Embolic material migration is the predominant risk factor associated with persistently worsening neurological outcome after combined treatment for challenging intracranial and facial tumors. To avoid such complications, both thorough knowledge of vascular anatomy and procedural ingenuity are essential.

Preoperative embolization remains as a supplementary treatment option, yet consensus on its effectiveness is absent. Therefore, we emphasize the responsibility of striving for safe procedures without an undue focus on achieving an exhaustive embolic effect.
